# Low Pitched Voices Are Perceived as Masculine and Attractive but Do They Predict Semen Quality in Men?

**DOI:** 10.1371/journal.pone.0029271

**Published:** 2011-12-21

**Authors:** Leigh W. Simmons, Marianne Peters, Gillian Rhodes

**Affiliations:** 1 Centre for Evolutionary Biology, School of Animal Biology (M092), University of Western Australia, Crawley, Western Australia, Australia; 2 ARC Centre of Excellence in Cognition and its Disorders, School of Psychology, University of Western Australia, Crawley, Western Australia, Australia; Federal University of Rio de Janeiro, Brazil

## Abstract

Women find masculinity in men's faces, bodies, and voices attractive, and women's preferences for men's masculine features are thought to be biological adaptations for finding a high quality mate. Fertility is an important aspect of mate quality. Here we test the phenotype-linked fertility hypothesis, which proposes that male secondary sexual characters are positively related to semen quality, allowing females to obtain direct benefits from mate choice. Specifically, we examined women's preferences for men's voice pitch, and its relationship with men's semen quality. Consistent with previous voice research, women judged lower pitched voices as more masculine and more attractive. However men with lower pitched voices did not have better semen quality. On the contrary, men whose voices were rated as more attractive tended to have lower concentrations of sperm in their ejaculate. These data are more consistent with a trade off between sperm production and male investment in competing for and attracting females, than with the phenotype-linked fertility hypothesis.

## Introduction

There is considerable evidence that women perceive masculine traits in men as attractive. Men with masculine faces [1], [2] and bodies [3], [4] are often rated as more attractive than their less masculine peers. Women's preferences for masculinity extend into the acoustic domain where a low voice pitch (the perceptual correlate of fundamental frequency) is rated by women as being masculine and attractive [5], [6]. Men with attractive voices tend also to have attractive faces [7], and the strength of women's preferences for masculine voices is positively correlated with their strength of preference for masculine faces [8], a correlation that can be explained by the structural connectivity between voice- and face-recognition areas of the brain [9]. Preferences for masculine voices have been found to vary across the menstrual cycle, peaking at the fertile phase [10], and to depend on relationship context, with a stronger preference for masculine voices when rating in the context of a short-term partner [11], [12]. Voice attractiveness has also been found to correlate with men's self reported number of sexual partners in North American populations of students [13], [14], and in a natural fertility population of hunter-gatherers, the Hadza of Tanzania, men's voice pitch was found to be a predictor of the number of living offspring fathered [15]. Collectively these findings suggest that sexual selection via female choice may have played a role in the evolution of sexual dimorphism in human voices. The aim of our study was to determine whether voice pitch might convey biological information relating to men's fertility, information that could provide women with direct benefits from mate choice.

Finding a fertile partner is an important step in successful reproduction. The phenotype-linked fertility hypothesis proposes that females can obtain reliable information about male fertility from the expression of their secondary sexual traits [16]. There have been reports of positive associations between secondary sexual trait expression and semen quality in non-human animals. In red deer, *Cervus elaphus*, for example, males with larger and more complex antlers also have larger testes and produce sperm with greater swimming velocity [17], a measure of ejaculate quality that predicts functional fertility [18]. Likewise, male stalk-eyed flies, *Teleopsis dalmanni*, with wider eye spans have larger testes and reproductive accessary glands, and females mated to these males exhibit greater fertility [19] [for other examples see 20,21,22,23]. For humans, Soler et al. [24] reported a positive association between facial attractiveness and semen parameters (sperm motility, morphology and concentration) in a sample of 66 Spanish men, although Peters et al. [25] were unable to replicate this finding with a sample of 118 Australian men, and found no relationship between semen quality and three components of facial attractiveness; averageness, symmetry and masculinity. No studies have examined the relationship between voice pitch and men's fertility, though such a relationship could in theory account for the finding that voice pitch predicts offspring production among Hadza men [15].

There are mechanistic reasons to think that voice pitch and semen quality might be linked. The development of secondary sexual traits is generally associated with increased levels of androgen hormones and in particular testosterone, in both non-human vertebrates [26], [27], [28], [29] and humans [30]. Elevated testosterone at puberty also stimulates an increase in the length of the vocal folds and a disproportionate growth of the larynx that together give men's voices their lower pitch [31], [32]. Moreover, there are androgen receptors associated with the vocal folds [33], [34], and circulating levels of testosterone are negatively correlated with voice pitch in adult men [35], [36], [37]. Androgen hormones also trigger testes maturation and sperm production, and testosterone levels in the testes regulate sperm production [38]. Testosterone thereby provides a mechanistic link between voice pitch and sperm production that could in theory provide an avenue for adaptive female choice [39], [40].

However, in contrast to the phenotype-linked fertility hypothesis, some studies have reported negative relationships between sexual trait expression and semen quality [41], [42]. Theoretical models of sperm competition have assumed that males face a resource allocation trade-off between acquiring mates and gaining fertilizations, such that males who invest heavily in competing for access to females have fewer resources available for sperm production [43]. There is some empirical support for this hypothesis. For example, in the field cricket, *Teleogryllus oceanicus,* males that invest in energetically expensive acoustic signals, those with high duty-cycles that are preferred by females, have lower sperm quality than males who produce less attractive though energetically cheaper courtship songs [42]. Likewise, male haubara bustards, *Chlamydotis undulata,* who engage heavily in extravagant behavioral and acoustic sexual displays suffer a reduction in spermatogenic function compared with males who invest less in such displays [44].

Our aim in this study was to determine whether masculinity in men's voices could provide cues to their fertility that females might use in choosing a high quality partner. To address this question we examined the relationship between men's voice pitch, women's perceptions of voice attractiveness and masculinity, and men's semen quality in a sample of Australian men. We predict that low pitched voices will be perceived as masculine and attractive, as found previously. The phenotype-linked fertility hypothesis predicts that voice attractiveness will be positively associated with men's semen quality. In contrast, a negative association between voice attractiveness and semen quality is predicted if men face a trade-off between attracting females and gaining fertilizations.

## Methods

This work was approved by the UWA Human Research Ethics Committee (Project number: 1074). All participants were provided with an information sheet outlining their role in the study, and were required to provide written consent.

### Participants and procedures

Fifty-four male participants (mean ± SE age 22 ± 0.5, range 18–32) were recruited by advertisement from the campus of the University of Western Australia. To ensure their anonymity, participants chose a 4 digit PIN with which to annotate all documents and samples that they would be required to provide for the study. All males were heterosexual and caucasian.

Voice recordings were made of the participants saying the vowels a, e, i, o, and u. Vowels were voiced at an interval of 1 per second, and recorded in an anechoic room using a Marantz PMD660 Professional digital recorder via a Røde NTG2 condenser microphone. Sampling frequency was 48 Hz and sampling depth 16 bit. Recordings were saved as WAV files.

Each participant completed a questionnaire regarding lifestyle factors that had the potential to impact their semen quality [25]. The questionnaire asked about age, weight and height, current medications, activity patterns including amount of exercise, alcohol and caffeine consumption, illicit drug use, dietary habits, frequency of sexual activity, and potential exposure to xenobiotics. The questionnaire is available from the authors on request.

Participants were given clear written instructions for the collection of a semen sample. They were asked to abstain from sexual activity for a minimum of 48 h and a maximum of 6 days prior to providing the sample. Semen quality can depend on the context in which the ejaculate is collected [45], [46]. Men were thus provided with the same set of 4 sexually explicit images, and asked to view these images immediately before collecting their semen sample. Semen was collected at home, by masturbation into a sterile vial. Vials were wrapped in insulating foil to maintain temperature, and delivered to the laboratory within 1 h of collection. Participants were asked to complete a second questionnaire to be returned with the sample, which noted the time of ejaculation, the time since their previous ejaculation, and the estimated proportion of the ejaculate captured in the vial. Finally, they were asked to return self-measured testes dimensions using disposable vernier calipers. Participants were provided with explicit pictorial instructions on how to measure the length and width of both left and right testes, from which volume could be estimated using the formula for an ovoid [4/3 × *π* × (length/2) × (width/2)^2^]. This procedure is highly repeatable and provides good estimates of testes size [47].

### Semen analysis

Semen samples were analysed immediately on delivery to the laboratory, using the Hamilton-Thorne CEROS Computer Assisted Semen Analysis (CASA) system. Samples of 2 µl were loaded into the chambers of a Leja Standard Count 4-chamber slide for analysis. We recorded the total concentration of sperm cells, and 7 motility parameters: average path velocity (VAP), straight line velocity (VSL), velocity along the sperm cells point-to-point track (VCL), the lateral amplitude of sperm head movement (ALH), the frequency with which the sperm head crosses the average sperm path (BCF), the straightness of the sperm's path (STR), and the linearity of the sperm's path (LIN). Sperm concentration was log transformed to achieve normality of distribution. The contributions of each of these semen parameters to male fertility are shown by Donnelly et al. [48] and Hirano et al. [49].

We summarized variation in the inter-correlated sperm motility scores using Principal Components Analysis [24], [25], [50]. The analysis returned three axes of variation (PCs) with eigenvalues >1.0, that collectively explained 94% of the variation in motility parameters (Table 1). PC1 was weighted most strongly by variables describing rapid progressive motility, PC2 was weighted most strongly by variables describing the directness or straightness of sperm tracks, and PC3 was weighted most strongly by the beat frequency of sperm heads.

**Table 1:** Means (±SE) of the sperm parameters, and the principal components analysis of their variation.

	Mean (±SE)	PC1	PC2	PC3
Eigenvalue		3.023	2.556	1.010
% variance explained		43.2	36.5	14.4
VAP *µ*m/s	54.6±1.6	0.506	0.286	0.065
VSL *µ*m/s	46.8±1.4	0.437	0.387	0.156
VCL *µ*m/s	75.4±2.2	0.548	0.101	−0.231
ALH *µ*m	4.7±0.1	0.374	−0.300	0.376
BCF beats/s	14.1±0.2	−0.047	0.382	−0.735
STR %	83.5±0.7	−0.257	0.510	0.239
LIN %	62.6±1.1	−0.211	0.512	0.422

VAP, average path velocity; VSL, straight line velocity; VCL, curvilinear velocity; ALH, lateral amplitude of sperm head; BCF, cross beat frequency; STR, straightness; LIN, linearity.

Semen quality can depend strongly on environmental factors, as well as procedural factors associated with sample collection [51], [52]. Therefore, we conducted a quality assessment of our semen data to account for any potentially confounding variables that might impact our analyses. We ran separate General Linear Models for sperm concentration, and for each motility PC, entering lifestyle factors (alcohol consumption, cigarette use, frequency of sexual activity, etc.) and procedural variables (time since last ejaculation, time from ejaculation to analysis, amount of ejaculate collected) as predictor variables. We also entered participant age, weight, height and combined testes volume as predictors. We then adopted stepwise elimination of non-significant terms as recommended by Crawley [53]. The only factor found to have a significant influence on our measures of sperm motility was the effect of the amount of ejaculate collected on PC1 (*F*_1, 52_ = 14.85, *P*<0.001); the greater the proportion of ejaculate collected the greater the progressive motility score. Early fractions of the human ejaculate contain prostatic components of the seminal fluid while later fractions contain components derived from the seminal vesicles, both of which contribute to the motility of ejaculated sperm [54], [55]. Failure to capture the entire ejaculate would thereby compromise motility. We therefore control for the proportion of ejaculate collected in all further analyses of PC1. Both the frequency of sexual activity and the period of abstinence prior to ejaculate collection influenced the concentration of sperm cells in the sample; participants who reported infrequent sexual activity (one or fewer ejaculations per week compared with 2, 3, or 4+ per week; *F*_4,48_ = 5.81, *P*<0.001), and those with longer periods of abstinence prior to sample collection (F_1,48_ = 4.26, P = 0.044) had higher sperm concentrations. We therefore controlled for variation in these variables in our analyses of sperm concentration.

### Voice analysis

Voice recordings were analyzed using the free voice analysis software PRAAT version 5.2.35 [56]. For each voice recording the pitch of each of the first four vowels ("a", "e", "i", & "o") was extracted, and an average pitch calculated across the four vowels. The vowel "u" was not included in the analysis because of the tendency for participants to intonate this vowel with a downward inflection. PRAAT calculates pitch using a noise-resistant autocorrelation method. We used PRAAT's standard settings: pitch floor of 75 Hz and ceiling of 600 Hz, window length 0.04 s and time step of 0.01 s. There were no significant relationships between voice pitch and men's height (*r* = −0.027, *P*  =  0.850), weight (*r*  =  0.018, *P*  =  0.896), testes volume (*r*  =  0.031, *P*  =  0.826), or age (*r* = −0.167, *P* = 0.227) so these variables were not considered further.

### Voice ratings

Thirty caucasian heterosexual females aged between 18 and 30 were recruited from the campus of the University of Western Australia to provide ratings of voice attractiveness and masculinity. Half the participants rated attractiveness and half rated masculinity. Voices were rated on a scale of 1(not attractive/masculine) to 10 (very attractive/masculine). Raters listened to the recordings through headphones, and rated each voice at the end of its presentation using the number keys on a computer keyboard (zero labeled as "10"). Voice recordings were presented using Superlab 4.0 and the order of presentation was randomized for each rater. Inter-rater agreement was very high, both for ratings of attractiveness (Chronbach's alpha 0.823) and masculinity (Chronbach's alpha 0.907). Average ratings of attractiveness and masculinity for each voice were calculated across raters and used in our analyses.

## Results

Sperm concentrations varied from 5×10^6^ sperm/ml to 658×10^6^ sperm/ml, with two participants having concentrations below the World Health Organization [52] lower threshold (15×10^6^ sperm/ml) for normal semen, and at the lowest centile expected for a general population [57]. Exclusion of these participants made no difference to the outcome of our analyses so they are retained in the results reported here. The mean (±SE) sperm concentration was 120.2±17.0 million sperm/ml. The mean sperm motility parameters for the participants in our sample are provided in Table 1. Values were within the ranges expected for normal semen [50].

**Figure 1:**
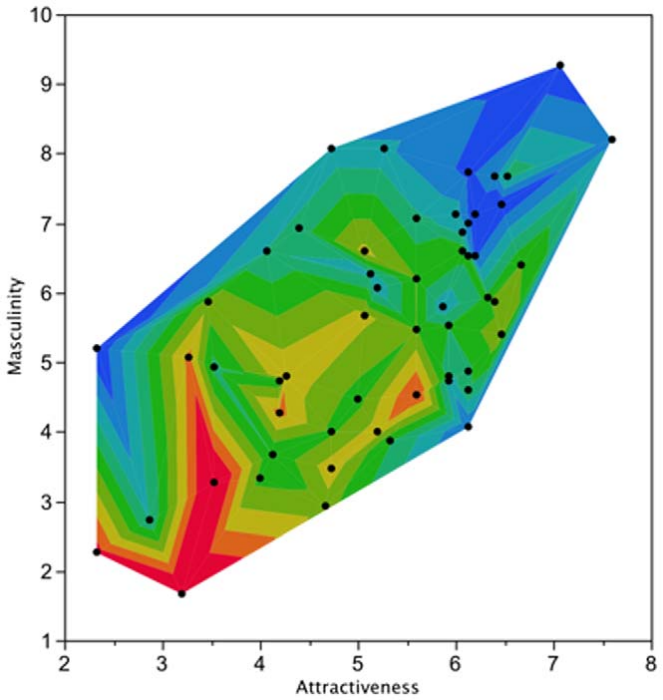
Contour plot showing the correlations between voice pitch, rated masculinity and attractiveness (colour "heat" corresponds to increasing voice pitch, which ranged from 85.3–134.2 Hz, blue being low pitch and red being high pitch).

**Figure 2:**
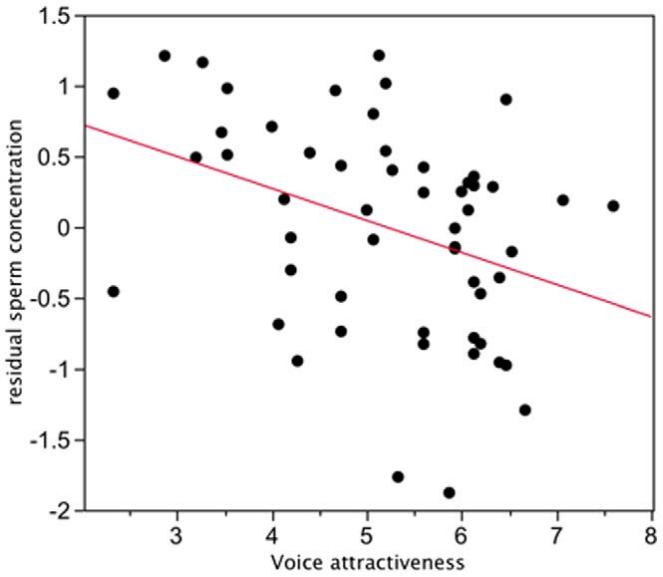
Scatterplot showing the association between voice attractiveness and sperm concentration (controlling for time since last ejaculation and frequency of sexual activity; red line shows the best least squares fit to the data).

The mean pitch of the participants voices was 105.6±1.6 Hz (range 85.3–134.2 Hz). Women rated voices of low pitch as being more attractive and more masculine than voices of high pitch, and masculine voices were also rated as being more attractive (Table 2, Fig. 1). Men with more attractive voices had lower sperm concentrations than men with less attractive voices. Although *P*  = 0.006, this relationship was not significant at the table-wise Bonferroni adjusted critical probability of *P*  = 0.003 (Table 2). Bonferroni adjustment is highly conservative, and there is a growing awareness that effect sizes and their confidence intervals are a more appropriate means by which to judge biological significance [58]. The correlation was of moderate size and the 95% confidence intervals suggest the effect of attractiveness on sperm count could range from a small to large effect (−0.114 to −0.580), but that an effect size of zero could be rejected with greater than 95% confidence. The correlation was not weighted by one or a few outliers, with data distributed evenly across the ranges of voice attractiveness and sperm concentration (Fig. 2). There were no significant correlations between men's sperm motility parameters and voice pitch, rated voice attractiveness, or rated voice masculinity (Table 2).

**Table 2:** Pearson correlations between voice parameters and semen quality.

	Attractiveness	Masculinity	Pitch
Masculinity	** 0.610		
Pitch	** −0.438	** −0.626	
Sperm/ml^1^	* −0.370	−0.175	0.203
Motility PC1^2^	−0.088	−0.039	0.001
Motility PC2	−0.001	0.064	−0.054
Motility PC3	−0.018	0.133	−0.053

1 correlations controlling for frequency of sexual activity and abstinence prior to sample collection.

2 correlations controlling for proportion of ejaculate collected.

*P = 0.006.

**P<0.001; the table-wise Bonferroni adjusted *P*_0.05_ = 0.003; N = 54.

## Discussion

Consistent with previous studies of voice attractiveness, we found that lower pitched voices were rated by women as being attractive and masculine [5], [6], [59], [60] giving our study external validity. Contrary to the phenotype-linked fertility hypothesis [16], men with attractive voices did not have better semen quality. Indeed the relationship between voice attractiveness and an important aspect of semen quality for men's fertility, sperm concentration [61], was negative, consistent with a potential trade-off between male expenditure on attracting females and gaining fertilizations.

This is one of only a handful of studies to explore a potential link between male attractiveness and reproductive health or fertility. Previously Soler et al. [24] reported a positive relationship between facial attractiveness and semen quality in a sample of Spanish men, a relationship that could not be replicated in a large sample of Australian men [25]. Measures of body asymmetry have been found to predict men's semen quality, with asymmetrical men having poorer semen quality than their symmetrical peers [62], [63]. The evidence that women can perceive subtle differences in body symmetry is mixed; some studies have shown an effect of body asymmetry on ratings of attractiveness while others have not, and the general effect from meta analysis of body symmetry on attractiveness is certainly weaker than it is for facial symmetry and attractiveness [64]. Interestingly, a significant and reasonably large positive association has been reported between voice attractiveness and body symmetry [65], implying that the voice could provide cues to men's reproductive health via the latters association with body symmetry. However, none of the semen parameters measured in our study were positively associated with voice attractiveness. More generally, studies that have looked for relationships between general health and attractiveness in face or body traits have yielded mixed results [66], [67]. For example, the mean general effect size for the relationship between symmetry and health appears to be in the region of 0.1, but varies considerable across studies from 0.08 to 0.67 [68]. Replicated studies such as ours are therefore valuable for gaining a better consensus view. This is the first study to have examined the relationship between voice attractiveness and an aspect of health, and we hope it will encourage further efforts in this area.

Our data showed that men with attractive voices had a lower concentration of sperm in their ejaculates. Animals have finite resources to partition amongst reproductive activities, and theoretical models of sperm expenditure assume a basic trade-off between male investment in attracting mates and in gaining fertilizations [43]. Recent studies of non-human animals are providing empirical evidence for this basic life-history trade-off [42], [44], [69], [70]. A number of studies have also reported short term declines in semen quality associated with social dominance. In domestic fowl, *Gallus gallus domesticus*, and arctic charr, *Salvelinus alpinus*, for example, males becoming dominant after a social challenge show a reduction in semen quality, while in cockroaches, *Nauphoeta cinerea*, both dominant and subordinate individuals suffer a reduction in ejaculate sperm counts resulting from the establishment of dominance hierarchies [71]. Thus, in non-human animals, there is evidence that males trade off investment in ejaculate quality when competing for and attracting mates.

In addition to being perceived as attractive, men with low pitched voices are also judged to be stronger, larger, better fighters and providers, and more dominant [5], [60], [72], [73], [74], and these judgments have been found to hold reasonable validity within western and hunter-gatherer societies [75]. The negative impact on semen quality of men's expenditure on physical training is well documented, where extreme investments in physical strength have been shown to affect the hypothalamus-pituitary-testes axis [76], [77], [78]. It is thus possible that investments in traits that contribute to dominance as well as attractiveness may come at the cost of reduced semen quality. Circulating levels of testosterone are associated with decreased voice pitch [35], [36], [37], increased masculine facial features [79], increased dominance [80], and men's success in obtaining sexual partners [81]. Although testosterone is required within the testes to regulate spermatogenesis [38], high levels of circulating testosterone can impair sperm production. Indeed, testosterone supplementation has been studied as a potential male contraception because of its negative effects on sperm production [82], with increased male aggressiveness noted as a problematic side effect [83]. Thus, elevated levels of testosterone associated with male attractiveness and dominance could suppress sperm production, mediating a negative relationship between these traits.

Although significant, the effect size for the association between sperm concentration and voice attractiveness was small. Moreover, sperm concentrations were largely within the range expected for functional fertility [52], so that women's preferences for men with attractive voices are unlikely to have implications for their ability to conceive. Nevertheless, even slight differences in semen quality can have considerable impact on competitive fertilization success; for example sperm velocity in fish [84], [85] and frogs [86], [87], and sperm viability in crickets [88], [89], have strong effects on fertilization success under competitive conditions, but these traits have little impact on male fertility in monogamous pairing. Thus, a weak phenotypic trade-off between attractiveness and sperm concentration is expected to have greater biological relevance in ancestral populations or natural fertility populations were females exercise polyandry. Finally we note that although in the same direction, men with low pitched voices tended to have a lower sperm concentration, this direct association was not significant. This may be due to lower statistical power, given the smaller effect size, or it may be that some parameter other than pitch also contributes to voice attractiveness and contributes to the trade-off with sperm concentration. Indeed, Feinberg et al. [90] have recently shown that both fundamental (pitch) and formant frequencies are integrated in women's preferences for men's voices. While pitch is determined by vibration of the vocal chords, formant frequency is determined by the resonant frequency of air in the vocal tract [91]. Importantly, vocal chord and vocal tract lengths are both influenced by testosterone [31], [32]. We therefore see our findings as preliminary, and argue that further study of the potential life-history trade-off between human mate attraction and reproductive health will prove fruitful.

In conclusion, our data support the view that women perceive men with low pitched voices as masculine and attractive. However, we find no support for the phenotype-linked fertility hypothesis. On the contrary, our data suggest a potential trade-off between men's attractiveness and sperm production that warrants consideration in future research.
